# Draft Genome Sequence of *Schnuerera* sp. Strain xch1, Isolated from the Tibetan Plateau

**DOI:** 10.1128/MRA.00993-21

**Published:** 2021-12-02

**Authors:** Jiahao Gu, Chenhan Xiao, Yongqin Liu

**Affiliations:** a State Key Laboratory of Microbial Metabolism, Shanghai Jiao Tong University, Shanghai, China; b Nord Anglia Chinese International School, Shanghai, China; c Center for Pan-Third Pole Environment, Lanzhou University, Lanzhou, China; d State Key Laboratory of Tibetan Plateau Earth System, Resources, and Environment, Institute of Tibetan Plateau Research, Chinese Academy of Sciences, Beijing, China; University of Delaware

## Abstract

In this study, we report the draft genome sequence of *Schnuerera* sp. strain xch1, which was isolated from a high-altitude thermal spring on the Tibetan Plateau. The enzymes and metabolic pathways of the strain may have further applications in biomass technology.

## ANNOUNCEMENT

The degradation of cellulose plays a key role in the cycling of carbon on our planet (1). Altitude is an important factor shaping the rate of cellulose decomposition (2). In the process of cellulose decomposition, cellobiose is a main product of cellulose degradation (1, 3). The β-glucosidase reaction that hydrolyzes cellobiose into two glucose monomers is considered to be the rate-limiting step in cellulose degradation to glucose (4). To study the enzyme activity of β-glucosidase from high-altitude sediments, a cellobiose-utilizing strain was isolated in this study.

The sediment sample was collected on 8 August 2019 from a low-temperature thermal spring with an altitude of 4,191 m, located in Sai Ma Chang (29°21′49.6″N, 96°52′11.2″E), a village of Ranwu county on the Tibetan Plateau. The temperature of the sediment sample was 13.1°C, and the pH was 8.56. For strain isolation, *Thermococcales* rich medium (TRM) (5) was modified before use. The salinity was adjusted to 1% (wt/vol) NaCl. The medium was adjusted to pH 7.5, autoclaved, and reduced to anaerobic conditions using 0.1% (wt/vol) sodium sulfide. The Hungate roll tube technique was used for strain isolation. The temperature for strain isolation was 42°C. A single colony was transferred into anaerobically modified TRM supplied with 1% (wt/vol) glucose and 1% (wt/vol) sulfur and was enriched at 42°C. A bacterial genome extraction kit (Sangon Biotech) was used to extract the genome. Library preparation was performed with the VAHTS universal DNA library preparation kit. The genome was sequenced with the Illumina HiSeq platform with 2 × 150-bp paired-end reads.

Sequencing produced 7,080,209 pairs of reads. Raw data were trimmed using Trimmomatic v0.36 (6) and Sickle v1.33 (https://github.com/najoshi/sickle) and assembled using SPAdes v3.15.2 (7). CheckM (8) was used to estimate the completeness of the genome. The genome was annotated using the NCBI Prokaryotic Genome Annotation Pipeline (PGAP) (9). Default parameters were used except where otherwise noted.

The 16S rRNA gene sequence placed strain xch1 within the genus *Schnuerera*, with 95.69% similarity to *Schnuerera ultunensis* DSM 10521 (GenBank accession number AZSU00000000.1) (10), which was isolated from an anaerobic sludge digester. The draft genome of *Schnuerera* sp. strain xch1, which has 45 contigs and a total length of 2,524,065 bp, was assembled. The *N*_50_ value is 116,266 bp. The G+C content of the genome is 31.23%. The genome contains 2,517 genes in total, with 2,443 protein-coding sequences. The genome encodes 43 tRNAs and 7 rRNAs, including 3 copies of 5S rRNA, 2 copies of 16S rRNA, and 2 copies of 23S rRNA. The completeness of the genome was estimated to be 99.3%.

Cellobiose can be used as a growth substrate instead of glucose. When supplied with 1% (wt/vol) cellobiose, the cell density can reach ∼10^7^ cells/ml, as measured with viable cell counts. No growth was observed when cellobiose or glucose was not added to the modified TRM. The isolation of *Schnuerera* sp. strain xch1 and the whole-genome sequencing give a possibility for further studies on cellobiose utilization through enzymes and metabolic pathways, which may have further applications in biomass technology.

### Data availability.

The genome of *Schnuerera* sp. strain xch1 and the raw sequence data are available in GenBank and the Sequence Read Archive (SRA) under the accession numbers JAIOUP000000000 and SRR15725293, respectively. The 16S rRNA gene sequence is available in GenBank under the accession number OK036738.
